# Extremohalophilic *Stutzerimonas stutzeri* mediated sodium complexation increases salt tolerance in wheat

**DOI:** 10.1080/15592324.2026.2716514

**Published:** 2026-08-14

**Authors:** Muhammad Zahid Mumtaz, Maham Rehman Khan, Azizullah Khalili, Latifa AlHusnain, Muneera D. F. AlKahtani, Mingming Wang, Naila Ali, Aveena Rehman Khan, Syed Zeeshan Haider Naqvi, Kotb A. Attia, Ahmed Mahmoud Ismail, Sajid Fiaz

**Affiliations:** a Northeast Institute of Geography and Agroecology, Chinese Academy of Sciences, Changchun, China; b Institute of Molecular Biology and Biotechnology, The University of Lahore, Lahore, Pakistan; c Department of Agronomy, Faculty of Agriculture, Sayed Jamaluddin Afghani University (SJAU), Kunar, Afghanistan; d Department of Biology, College of Science, Princess Nourah bint Abdulrahman University, Riyadh, Saudi Arabia; e Azra Naheed Medical College, Superior University, Lahore, Pakistan; f Center of Excellence in Biotechnology Research, King Saud University, Riyadh, Saudi Arabia; g Pests and Plant Diseases Unit, College of Agricultural and Food Sciences, King Faisal University, Al-Ahsa, Saudi Arabia; h Life Sciences Research Center, Western Caspian University, Baku, Azerbaijan

**Keywords:** Halophilic bacteria, salt tolerance, Ion balance, nutrient solubilization

## Abstract

**Background:**

Hypersaline-tolerant bacterial metabolites are believed to play a critical role in sodium detoxification and salt stress tolerance in plants; however, this mechanism needs further investigation. This study aimed to evaluate halophilic bacterial strains from hypersaline environments for their ability to increase salt tolerance in wheat seedlings through sodium‒organic acid complexation, nutrient dissolution, and improved ionic homeostasis.

**Methods:**

The halophilic bacterial strains were isolated from hypersaline conditions of salt mines and were characterized for multiple plant growth-promoting traits. These strains were tested on wheat seedling biomass, chlorophyll, osmolyte accumulation, antioxidant defense, and ion homeostasis under salt stress. The strains were assessed for their ability to produce organic acids and increase nutrient availability by solubilizing insoluble minerals under salt stress.

**Results:**

The isolated bacterial strains were salt-tolerant up to 2 M NaCl stress, solubilized insoluble minerals, and produced indole acetic acid, siderophores, ammonia, hydrogen cyanide, exopolysaccharides, and various enzymes. They were identified as *Stutzerimonas stutzeri* (strains MRK6 and MRK20) and *Pseudomonas aeruginosa* (strains MRK7 and MRK11). *S. stutzeri* MRK6 showed the highest increase in wheat seedling growth, chlorophyll, osmolytes accumulation, antioxidant enzymes, and ion homeostasis by increasing potassium uptake and modulating sodium toxicity under 100 mM salt stress. This increased nutrient availability from insoluble minerals.

**Conclusion:**

The halophilic *S. stutzeri* MRK6 increased salt tolerance in wheat by increasing soil mineral dissolution, sodium detoxification, and ionic compartmentalization rather than excessive sodium uptake. This mechanism offers a promising approach for mitigating salt stress in salt-affected soils.

## Introduction

Salt stress is a major ecological stressor that reduces agricultural productivity by degrading fertile land into marginal soils and decreasing crop yield.^1^ Over the last two decades, nearly 2000 ha of irrigated land in arid and semi-arid areas around the globe have been degraded by salinity in a single day.^2^ Recent estimations by FAO^3^ have shown that salt affects 20%–50% of irrigated soils globally. Crops grown in saline environments showed a reduction in crop yields and alterations in soil physicochemical properties, which disrupt the region's ecological balance.^4^ It reduced crop yield by limiting the uptake of nutrients and water and through intricate interactions among morphological, physiological, and biochemical processes.^4^ ^,^ ^5^ Salt stress affects nearly every stage of crop development through oxidative and osmotic stress, ion toxicity, and restricted nutrient uptake.^6^ Osmotic stress limits water availability, leading to dehydration in plants and a loss of cellular turgor.^7^ It affects plant growth and development by accumulating Na^+^ and Cl^−^ ions in plant tissues, leading to disturbances in mineral nutrition^4^ Consequently, secondary oxidative stress appears due to osmotic and ionic stress.^8^ It affects the photosynthetic processes by affecting the efficiency of photosystem II, reducing the leaf area, chlorophyll content, and stomatal conductance.^9^ Salt stress prevents protein synthesis, lipid metabolism, and physiological and molecular development in plants.^6^

The Khewra Salt Mine is the second-largest salt mine worldwide. It is situated in the foothills of the Salt Range in Punjab, Pakistan, and has a unique ecological niche where highly specialized plant species can survive.^10^ The extreme salinity and sodicity in this region's soil cause a harsh environment and impose significant osmotic and ionic stress on local plant species.^11^ Despite these adverse conditions, several halophilic plants have adapted to the saline and sodic landscape of the Khewra Salt Mine through their physiological and biochemical strategies to overcome salt stress.^11–13^ Halophilic bacteria are crucial for promoting plant survival in highly saline environments. They thrive in the rhizosphere and within plant tissues of native halophilic plants grown under high-salt environments.^14^ Over the last decade, researchers have highlighted the importance of halophilic bacteria in improving plant growth under high-salinity conditions.^15^ ^,^ ^16^

Halophilic plant growth-promoting bacteria (PGPB) develop symbiotic relationships with plants and regulate plants' ability to withstand salt stress. They colonize the plant rhizosphere and trigger multiple stress-alleviating pathways.^17^ Halophilic bacteria produce 1-aminocyclopropane-1-carboxylate deaminase, which reduces ethylene production and alleviates salt stress in plants.^18^ They produce indole-3-acetic acid (IAA), leading to extensive root systems that explore larger soil volumes to uptake water and nutrients under saline conditions.^19^ ^,^ ^20^ Bacteria-derived IAA promotes root development by stimulating cell division, elongation, and lateral root formation, resulting in an improved root system that enhances water and nutrient uptake under saline conditions. Consequently, these bacteria can contribute to improved plant growth and salt stress adaptation by maintaining root biomass.^21^ Halophilic bacteria can solubilize insoluble phosphate and micronutrient minerals into plant-accessible forms.^22^ ^,^ ^23^ These bacterial traits increase plant growth in nutrient-deficient saline soils around the Khewra Salt Mine, where nutrient availability is disrupted due to ionic toxicity. Halophilic bacteria mitigate oxidative stress by triggering plant antioxidant defense systems and promoting the activities of various antioxidant enzymes to scavenge reactive oxygen species (ROS).^24^ They mitigate osmotic stress by promoting the accumulation of osmoprotectants in plant cells and stabilizing proteins. Such bacteria can sequester toxic Na^+^ and Cl^−^ ions through their ion transport system. They maintain cellular homeostasis in the host plant by reducing ion toxicity.^25^ The symbiotic association between halophilic bacteria and plants under salt stress represents an adaptive survival approach shaped by extreme environmental pressures. These beneficial microbes provide a wide range of support, from hormonal regulation and nutrient acquisition to antioxidant defense and ion homeostasis, which helps plants to survive under extremely saline conditions.^26^ Halophilic bacteria have gained attention for their potential in mitigating salt stress in plants. These microorganisms possess various plant growth-promoting traits, which enable them to enhance plant growth under saline conditions.^27^ ^,^ ^28^

Halophilic bacteria are promising biotechnological resources for sustainable agriculture, particularly in developing microbial strategies to improve crop productivity in salt-affected soils. In this study, we hypothesized that multifunctional plant growth-promoting halophilic bacterial strains from a hypersaline environment can mitigate salt stress in wheat seedlings by increasing nutrient availability and promoting Na^+^ detoxification and sustaining ionic homeostasis. To test this hypothesis, we evaluated halophilic plant growth-promoting bacterial strains from the hypersaline ecosystem of Khewra Salt Mines to increase wheat seedling growth, photosynthesis, osmolyte accumulation, antioxidant defense, and ion homeostasis under salt stress. Unlike previous studies that have focused largely on conventional saline environments, this work investigated hypersaline-tolerant bacterial strains from underexplored brine ponds and halophyte-associated root zones. It revealed their potential role in Na^+^ detoxification, mineral dissolution, and stress adaptation. Particularly, isolated bacterial strains showed exceptional halotolerance, surviving up to 2 M NaCl, exceeding the 1.5 M tolerance reported in previous studies.^29^ ^,^ ^30^

## Materials and methods

### Sample collection

The saline water samples were collected from a brine pond in the Khewra Salt Mine, Pakistan, at 32.65°N latitude, 73.01°E longitude, and 285 m elevation. Rhizospheric soil samples were collected from *Muhlenbergia rigens,* which are widely found in the foothills of the Khewra Salt Mine in Pakistan. This mine is located about 100 miles from Islamabad and Lahore. The mine is in the Salt Range and rises from the Indo-Gangetic plain. Globally, it is the second-largest salt mine and is famous for its Himalayan salt production. Sterile plastic containers were used for the collection of saline water and rhizospheric soil samples, and transported to the laboratory under low temperatures in a cooler box. The collected saline water and rhizospheric soil sample pH were 9.9 and 9.1, respectively, while electrical conductivity was 211 dS m^−^^1^ and 6.24 dS m^−^^1^.

### Isolation of halophilic bacteria

The collected samples were serially diluted in autoclave-distilled water. The autoclaved Luria–Bertani (LB) agar medium, amended with 1 M NaCl, was solidified into sterilized Petri dishes. The diluted samples were inoculated onto agar plates using a sterile spreader and incubated at 30 °C for up to 72 h. The well-separated colonies with different appearances were purified after several strikes on agar plates and preserved in glycerol (50%) at 4 °C and then −20 °C. The isolated colonies were tested for their halophilic nature by culturing in LB broth supplemented with 0.5, 1.0, 1.5, and 2.0 M NaCl levels. The cultures were incubated in a shaking incubator at 30 °C and 100 rpm for 96 h. After incubation, the cultures were read on a spectrophotometer at 600 nm to observe bacterial biomass production. Simultaneously, these cultures were subjected to a colony-forming unit (CFU) mL^−1^ by preparing dilutions up to 10^12^. Halophilic bacterial isolates with higher biomass, CFU, and higher salt tolerance were selected for further evaluation.

### Screening of halophilic bacterial strains for plant growth promotion

The halophilic bacterial strains were screened for their plant growth-promoting nature by conducting a jar experiment. Wheat seed variety Faker-e-Bhaker was surface sterilized with 0.2% MgCl_2_ and 95% ethanol for 3 min each and then extensively washed with sterile distilled water. The pure bacterial cultures were grown in LB broth for 48 h at 30 °C under shaking conditions. Surface-sterilized wheat seeds were soaked in broth media for one hour, and ten seeds were sown in plastic jars containing sterile sand. Initially, the nutritional and water requirements were fulfilled with a half-strength Hoagland solution, and afterward, tap water was used to meet the water requirements. The experiment was incubated in a growth room with 14 h of light and 10 h of darkness under a completely randomized design with five replications. Upon seed germination, five wheat seedlings were maintained in each jar. After three weeks of germination, the wheat seedlings were harvested, and data regarding shoot and root growth and biomass were taken to compare the growth promotion of wheat by halophilic bacterial strains compared to control plants.

### Characterization of plant growth-promoting traits

The halophilic bacterial strain's ability to produce indole compounds was determined using Salkowski's colorimetric technique^31^ under 100 mM NaCl. Bacterial cultures were grown in a nutrient broth medium containing 1% (w/v) L-tryptophan and 100 mM NaCl at 30 °C and 100 rpm for 72 h. These cultures were harvested through centrifugation at 10,000 × *g* for 5 min, and supernatant (1 mL) was mixed with Salkowski's reagent (2 mL). The reaction mixture was placed in the dark for 30 min and the OD was recorded at 530 nm. The indole compound concentration was calculated by plotting a standard curve. The negative control value was subtracted from the indole compound concentration of the bacterial strains. The atmospheric N_2_-fixation ability of halophilic bacterial strains was determined using N-free semi-solid malate medium at 30 °C for 72 h.^32^ The change in color from pale green to blue around the bacterial colonies were considered positive for nitrogen fixation. Siderophore production by halophilic bacterial strains was qualitatively determined through a disc diffusion chrome azurol sulfonate agar assay.^33^ A loopful of actively growing bacterial cells was spot-inoculated on chrome azurol sulfonate agar medium. After incubation at 30 °C for 72 h, the yellow‒orange zone around bacterial growth was considered siderophore positive. To determine ammonia production by halophilic bacterial strains, freshly grown cultures were inoculated in test tubes containing peptone water and incubated at 30 °C for 72 h. After incubation, the cultures (1 mL) were reacted with Nessler's reagent (50 μL), and the presence of a faint yellow to brownish color was considered ammonia-positive.^34^ ^,^ ^35^ Owing to high salt concentrations interfering with colorimetric measurements, these results were interpreted as comparative rather than absolute ammonia quantification.

A method by Bakker and Schippers^36^ was adopted to monitor the HCN trait in halophilic bacterial strains. Fresh cultures were streaked on LB agar containing glycine (4.4 g L^−1^) and placed at 30 °C for 24 h. After that, the Petri dish cover was inverted, and autoclaved filter papers soaked in a reagent composed of 0.5% picric acid and 2% Na_2_CO_3_ were placed inside the Petri dish covers. The Petri dishes were sealed with parafilm and kept at 30 °C for 72 h. The strains were considered HCN-positive by observing the filter paper's color change from orange to red. EPS by halophilic bacterial strains was determined by inoculating fresh inoculum on RCV agar media, and the appearance of mucoid growth was considered EPS-positive.^37^ The halophilic bacterial strain's ability to utilize citrate as its carbon and energy source was tested using a Simmons citrate slant medium. The appearance of green was considered positive for citrate metabolism.^38^

### Enzymatic activities of halophilic bacterial strains

The halophilic bacterial strains were tested for various enzymatic activities by adopting the standard protocol of Cappuccino and Sherman.^34^ Fresh bacterial strains were grown as a single streak on starch agar for 72 h at 30 °C for amylase activity. The media surface was flooded with iodine solution with a dropper for 30 s, and the excess iodine was rinsed. The development of clear, transparent halos was considered a sign of bacterial strains positive for amylase activity to hydrolyze starch. For catalase activity, freshly grown bacterial cells were placed on clean microscopic slides and reacted with one drop of H_2_O_2_. Air bubble development was recorded in catalase-positive halophilic bacterial strains. The bacterial strains were grown on carboxymethyl cellulose agar media for cellulose activity and incubated at 30 °C for 72 h. Further, the media were flooded with an iodine solution, and a halo zone around the bacterial colonies was observed. For lipase activity, bacterial cells were streaked on a medium composed of 2.0% olive oil (v/v), 1.0% Tween 80 (v/v), and 0.001% Rhodamine B (w/v). After 72 h of incubation, the halo zones around the bacterial growth were recorded as positive for lipase activity. The bacterial cultures were grown on 1% skim milk agar, and the clearance zone around the bacterial growth was recorded as positive for protease activity. Freshly grown bacterial cells were treated with Kovacs oxidase reagent, and the appearance of a deep-purple hue was considered an oxidase-positive bacterial strain. Urease-based broth was inoculated with bacterial strains and incubated for 48 h at 30 °C to observe the development of a yellow-to-pink color. The safety of tested bacterial strains was tested by performing a coagulase test. A drop of plasma was placed on a microscopic slide and mixed with a loop full of bacterial cells. After 10 s, the slides were analyzed for clumping as a coagulase activity.

### Determination of mineral dissolution by halophilic bacterial strains

The selected plant growth-promoting halophilic bacterial strains were tested for the dissolution of insoluble minerals to increase the availability of phosphorus (P), potassium (K), zinc (Zn), and manganese (Mn) for plant uptake. For P dissolution, Pikovskaya's agar was amended with tricalcium phosphate (0.5%) and 100 mM NaCl. For K dissolution, Aleksandrov medium with potassium aluminum silicate (0.2%) and 100 mM NaCl was spot inoculated with bacterial cells. Tris-minimal salt agar medium amended with zinc oxide (0.1%) and 100 mM NaCl was inoculated with bacterial cells to test Zn dissolution. The nutrient agar medium was amended with 50 mM manganese oxide and 100 mM NaCl for Mn dissolution, and the bacterial cells were spot-inoculated. All these experiments were incubated at 30 °C for five days in three replicates, and the appearance of the halo diameter was recorded. In the case of K and Mn dissolution, the iodine solution was flooded in an agar medium for five minutes, and the extra liquid was rinsed. The phosphate dissolution diameter (PSD), potassium dissolution diameter (KSD), zinc dissolution diameter (ZSD), and manganese dissolution diameter (MSD) were estimated using ImageJ software. The formula reported by Ahmad et al.^39^ was used to determine the phosphate dissolution index (PSI), phosphate dissolution efficiency (PSE), potassium dissolution index (KSI), potassium dissolution efficiency (KSE), zinc dissolution index (ZSI), zinc dissolution efficiency (ZSE), manganese dissolution index (MSI), and manganese dissolution efficiency (MSE).

### Application of halophilic bacterial strains for alleviation of salt stress in wheat

A pot experiment was performed to assess the potential of halophilic bacterial strains to alleviate salt stress in wheat seedlings. Soil sieved through a 2 mm mesh was used to fill the plastic pots containing a hole at the bottom (11 cm in length, top diameter, and 6 cm in bottom diameter). The plastic pots were filled with 500 g of soil and moistened with a half-strength Hoagland solution. The physicochemical properties of the experimental soil were sandy loam, pH 8.1, electrical conductivity, 1.2 dS m^−1^, organic matter 0.35%, available nitrogen 0.11%, available phosphorus 6.03 mg kg^−1^, available potassium 130 mg kg^−1^, available zinc 3.01 mg kg^−1^, and available manganese 6.05 mg kg^−1^. This soil contained total phosphorus of 898.6 mg kg^−1^, total potassium of 1305.5 mg kg^−1^, total zinc of 49.7 mg kg^−1^, and total manganese of 395.4 mg kg^−1^. Most of the total nutrient concentrations were expected to be present in fixed/insoluble forms, with only a small fraction available to plants. The objective of this study was to solubilize these fixed nutrients by applying halophilic bacteria under salt stress conditions.

The pure colonies of halophilic bacterial strains were freshly grown in nutrient broth media in a shaking incubator. The prepared inoculum was used for inoculation in surface-sterilized wheat seeds. Wheat seeds (variety Faker-e-Bhaker) were purchased from a local market, surface sterilized with 0.2% mercuric chloride and 95% ethanol separately for 3 min and successively washed numerous times with sterile distilled water. Surface-sterilized seeds were soaked in bacterial cultures (10^7^ CFU mL^−1^) for one hour, and ten seeds from each bacterial culture were sown at three salt levels (0, 50, and 100 mM NaCl stress) based on mild to moderate salt stress.^40^ For the control group, seeds were soaked in a broth medium without bacterial growth and grown simultaneously along with other treatments. The experiment was arranged in a triplicate CRD design under natural weather conditions, and the experiment was protected from bird and animal attacks by placing it in a net house. After seed germination, salt stress of 0 mM, 50 mM, and 100 mM NaCl was prepared in tap water and used to irrigate the pots at a 33% saturation percentage of soil (v/w). Osmotic shock in wheat seedlings was prevented by allowing the seedlings to develop root systems for up to two weeks, and salt stress was gradually added at two split doses of 50 mM with a week's interval. The leached salt water was collected and again placed in their respective pots. A half-strength Hoagland solution facilitated the rest of the water and nutritional requirements. Wheat seedlings were harvested after two weeks of salt stress, and data regarding growth and biochemical traits were observed.

The seedling root and shoot lengths were measured using a meter rod. Their fresh biomass was taken soon after harvesting through an electrical balance. The seedlings were dried at 76 °C and weighed to record the dry weight. Leaf samples were extracted with 80% acetone to determine the total soluble sugar, phenols, amino acids, and protein contents. To determine the total phenolic contents, the leaf extracts (1 mL) were treated with Na_2_CO_3_ (5 mL) and Folin–Denis's reagent (1 mL). The mixture was shaken with the help of a shaker, and OD was taken at 765 nm.^41^ The total soluble sugar was determined by following the method of Riazi et al.^42^ The 0.5 mL fresh leaves extract was mixed with 2 mL anthrone and heated for 10 min at 80 °C. The mixer was cooled, and the total soluble sugar was determined by measuring OD at 620 nm. The method of Hamilton et al.^43^ was adopted to determine total free amino acids. The leaf extract (1 mL) was mixed with pyridine (10%) and ninhydrin (1%). After 30 min of incubation, the OD was read at 570 nm, and the free amino acids were determined. Total soluble protein was extracted and determined. The 1 mL of leaf extract was mixed with a 1 mL solution composed of sodium potassium tartrate and copper sulfate. After 30 min of incubation at room temperature, the OD was read at 620 nm to calculate the total soluble protein. The shoot samples were wet digested following the method of Wolf.^44^ The plant-digested samples were used to determine the nitrogen concentration through the Kjeldahl method, phosphorus concentration through the yellow vanadomolybdate reagent,^45^ and the potassium and sodium concentrations through a flame photometer.

### 16S rRNA partial gene sequencing

The four best plant growth-promoting halophilic bacterial strains, MRK6, MRK7, MRK11, and MRK20, were selected for 16S rRNA gene sequencing. The DNA of these bacterial strains was extracted and amplified using universal primers by following the same procedure as reported in our previous work.^46^ The purified PCR product was sequenced through a commercial service by Macrogen Seoul, Korea (https://dna.macrogen.com/). The forward and reverse sequences were used to generate consensus sequences using the software BioEdit Sequence Alignment Editor (version 7.2; https://bioedit.software.informer.com/7.2/). The consensus sequences were blasted in the EZBioCloud database using 16S-based ID (https://www.ezbiocloud.net/), and the target strain was identified using species and genus names of the closely matched type strain. The sequences were submitted to the NCBI Gene Bank (https://www.ncbi.nlm.nih.gov/genbank/submit/), and accession numbers MW69354 for strain MRK6, MW69355 for MRK7, MW69357 for MRK11, and MW69359 for MRK20 were obtained. The phylogenetic tree of the identified bacterial strains and closely related type strains was prepared using software MEGA version 11 through ClustalW alignment and bootstrap support values of 1000 replicates.

### Determination of organic acid production in response to a consortium of insoluble minerals under salt stress

The halophilic bacterial strain's ability to solubilize a consortium of insoluble minerals was determined using a modified Pikovskaya's broth medium. The medium was composed of glucose (10 g L^−1^), (NH_4_)_2_SO_4_ (0.5 g L^−1^), NaCl (0.2 g L^−1^), KCl (0.2 g L^−1^), MgSO_4_7H_2_O (0.1 g L^−1^), and yeast extract (0.5 g) amended with insoluble minerals, including tricalcium phosphate (0.5%), potassium aluminum silicate (0.2%), zinc oxide (0.2%), and manganese dioxide (50 mM). Additionally, NaCl of 100 mM concentration was added to the developed broth medium to create salt stress. The pH of the medium was adjusted to 7.0 using 1 N NaOH/HCl, and autoclaved at 121 °C for 20 min. The selected bacterial cultures of strains MRK6 and MRK7 were inoculated aseptically at 10^8^ CFU mL^−1^ and incubated at 30 °C for five days under shaking conditions at 120 rpm in triplicate. After five days of incubation, the pH of the cultures was determined. These cultures were centrifuged at 5000 × *g* for 10 min at 4 °C. The cultures were filtered through 0.45 µm membrane filters and were subjected to the determination of organic acid and available nutrients. The phosphorus concentration was determined through the yellow vanadomolybdate reagent,^45^ potassium through a flame photometer, and zinc and manganese were determined through an atomic absorption spectrophotometer. The culture filtrates were analyzed for organic acids through GC-MS (ISQ Trace 1300, Thermo Fisher Scientific, Canada) and HPLC (1100 Series, Agilent Technologies, Germany).

### Statistical analysis

The *in vitro* data set of plant growth-promoting traits was subjected to a one-way analysis of variance (ANOVA) under a linear model with a completely randomized design. The data set of the pot experiment under salt stress was subjected to a two-way ANOVA with the factorial model of a completely randomized design. The ANOVA was performed through the statistical software Statistix version 8.1 at 5% probability. The post-hoc least significant difference test was performed to differentiate the significance between different treatment means. The data was presented as a bar graph, a heat map, and a correlation matrix. The treatment means were compared through standard error and alphabetical letters. The means sharing the same alphabetical letters were considered statistically non-significant at *p* ≤ 0.05. A salt tolerance index was calculated from standardized physiological traits PCA-based weighting. Random forest regression was performed to identify the relative contribution of individual traits to salt tolerance index variation. The physiological traits were used as predictor variables, and the model was constructed with 5000 trees. Trait importance was assessed based on the percentage increase in the mean squared error (%IncMSE). Pearson correlation coefficients between individual traits and the salt tolerance index were calculated, and standardized z-values were used to visualize positive and negative trait contributions to salt tolerance. All the statistical analyses and visualizations were performed in R using relevant packages, including randomForest, ggplot2, and corrplot.

## Results

### Halophilic nature of isolated bacterial strains

**Figure 1.**
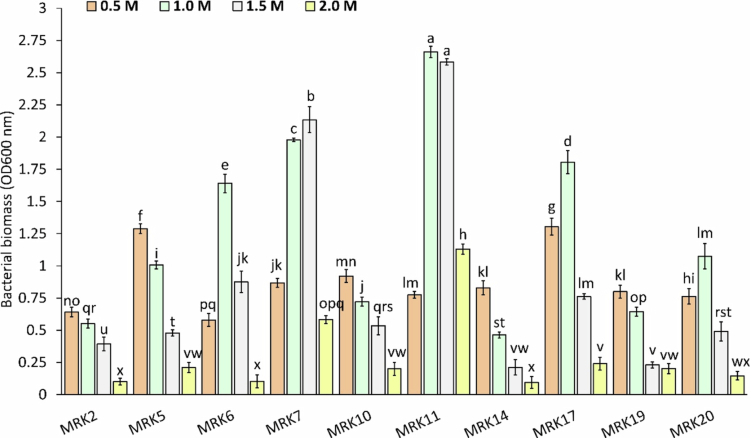
The bacterial biomass in the form of optical density at 600 nm of isolated bacterial strains from the brine ponds of the Khewra salt mine and rhizospheric soil of *Muhlenbergia rigens* under 0.5 M to 2.0 M salt stress; these bacterial strains demonstrated their halophilic nature by growing up to 2.0 M salt stress; the presented bar are mean values of three replications statistically analyzed using factorial design arrangements along with alphabetical letters and standard error; the bars with same alphabetical letters were considered statistically non-significant at *p* ≤ 0.05.

More than 20 bacterial strains capable of tolerating 250 mM NaCl were isolated from the brine pond of the Khewra Salt Mine, which has an alkaline pH of 9.9. Among these, three promising strains, MRK2, MRK5, and MRK6, were selected for further evaluation under higher salt concentrations, up to 2 M NaCl. In parallel, more than 30 bacterial strains capable of tolerating 250 mM NaCl were isolated from the rhizospheric soil of *M. rigens*, a halophytic grass naturally growing near the Khewra Salt Mine, where the soil pH was 9.1. Among these 30 bacterial strains, MRK7, MRK10, MRK11, MRK14, MRK17, MRK19, and MRK20 were further tested up to 2 M NaCl stress. The selected bacterial strains demonstrated their potential to grow under salt stress ranging from 0.5 M to 2.0 M (Figure 1). The strains MRK5 and MRK17 showed their maximum microbial biomass in 0.5 M salt stress. Their growth decreased with increasing salt stress, and they had the lowest microbial biomass at 2.0 M salt stress. The strains MRK7 and MRK11 had the highest microbial biomass at 1.0 M, 1.5 M, and 2.0 M salt stress. These findings indicate the hyperhalophilic nature of the bacterial strains, which were selected for further evaluation for plant growth.

### Plant growth-promoting ability of halophilic bacteria

**Figure 2.**
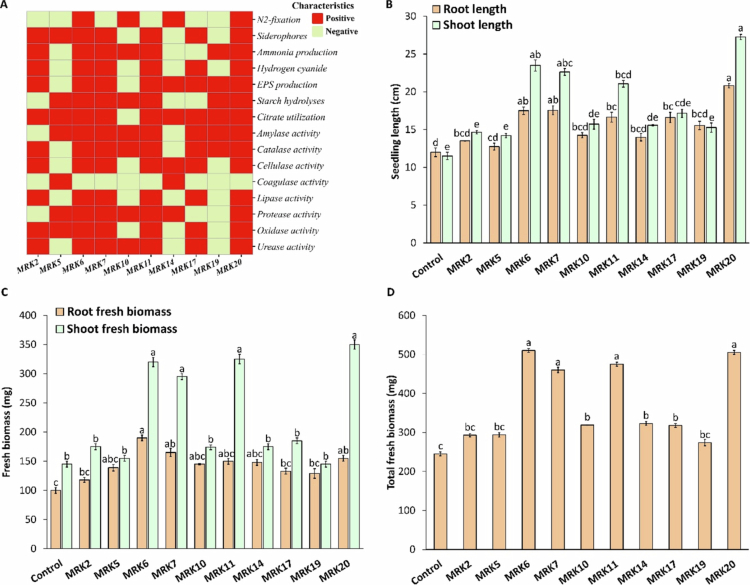
Halophilic bacterial strains demonstrated *in vitro* plant growth-promoting traits (a) and their ability to promote wheat root and shoot length (b), root and shoot fresh biomass (c), and total biomass (d) under anoxic conditions; the positive characteristics given in (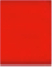) represents the presence of traits, and the negative characteristics given in (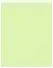) represent the absence of traits in tested halophilic bacterial strains; the presented bar in (b–d) are mean values of three replications statistically analyzed using factorial design arrangements along with alphabetical letters and standard error; the bars with same alphabetical letters were considered statistically non-significant at *p* ≤ 0.05.

The selected ten halophilic bacterial strains promoted wheat growth under anoxic conditions (Figure 2B–D). The wheat seedlings treated with halophilic bacterial strains had higher root length, shoot length, root fresh weight, shoot fresh weight, and total fresh biomass than the uninoculated control. The highest increase of 73% in root length was achieved due to inoculation with MRK20, followed by MRK6 (65%) and MRK7 (46%) compared to the uninoculated control. The wheat seedlings treated with MRK20, followed by MRK6 and MRK7, promoted shoot length to 137%, 104%, and 97% over the uninoculated control. The highest increase of 90% in root fresh weight was due to strain MRK6. The plants treated with MRK6, MRK7, MRK11, and MRK20 reported the highest increase in shoot fresh weight and total fresh biomass. These strains promoted shoot fresh weight ranging from 103% to 141% and total fresh biomass ranging from 88% to 106% over the uninoculated control.

### Halophilic bacterial strains possess multiple beneficial traits

**Figure 3.**
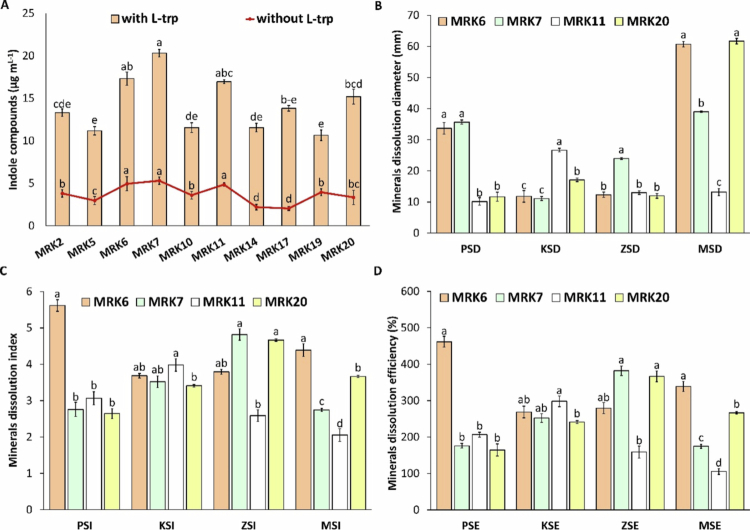
The halophilic bacterial strains produced indole compounds in the presence and absence of L-tryptophan under 100 mM NaCl stress (a) and demonstrated dissolution of insoluble minerals under 100 mM NaCl stress measured in minerals dissolution diameter (b), mineral dissolution index (c), and minerals dissolution efficiency (d); the indole compounds was determined through Salkowski's colorimetric technique by growing halophilic bacterial strains in nutrient broth amended with and without 1% L-tryptophan for 72 h under shaking conditions; Indole compounds values were calculated after subtracting values of negative controls; the dissolution of insoluble minerals, including tri-calcium phosphate, mica, zinc oxide, and manganese oxide were tested under agar plates containing their respective media; the presented bar graphs are mean values of three replications statistically analyzed using completely randomized design arrangements; the mean values were compared through alphabetical letters and standard error; the bars with same alphabetical letters were considered statistically non-significant at *p* ≤ 0.05; PSD, phosphate dissolution diameter; KSD, potassium dissolution diameter, ZSD, zinc dissolution diameter; MSD, manganese dissolution diameter; PSI, phosphate dissolution index; KSI, potassium dissolution index; ZSI, zinc dissolution index; MSI, manganese dissolution index; PSE, phosphate dissolution efficiency; KSE, potassium dissolution efficiency; ZSE, zinc dissolution efficiency; MSE, manganese dissolution efficiency.

The halophilic bacterial strains possess multiple *in vitro* beneficial traits, including nitrogen fixation; the production of siderophores, ammonia, HCN, polysaccharides, starch hydrolysis, and citrate utilization (Figure 2A). Among the tested strains, 70% were positive for the production of siderophores, ammonia, HCN, cellulase, protease, oxidase, lipase, and urease. All the strains were able to produce exopolysaccharides (except MRK5 and MRK10), citrate utilization (except MRK10), amylase activity (except MRK2 and MRK14), and catalase activity (except MRK5 and MRK14). The 40% of the halophilic strains showed nitrogen fixation, while only MRK5 and MRK14 demonstrated coagulase activity. The tested halophilic bacterial strains demonstrated *in vitro* indole compound production in the absence and presence of L-tryptophan under 100 mM NaCl (Figure 3A). Strain MRK7 reported the highest production of indole compounds in the absence and presence of L-tryptophan under 100 mM NaCl.

**Figure 4.**
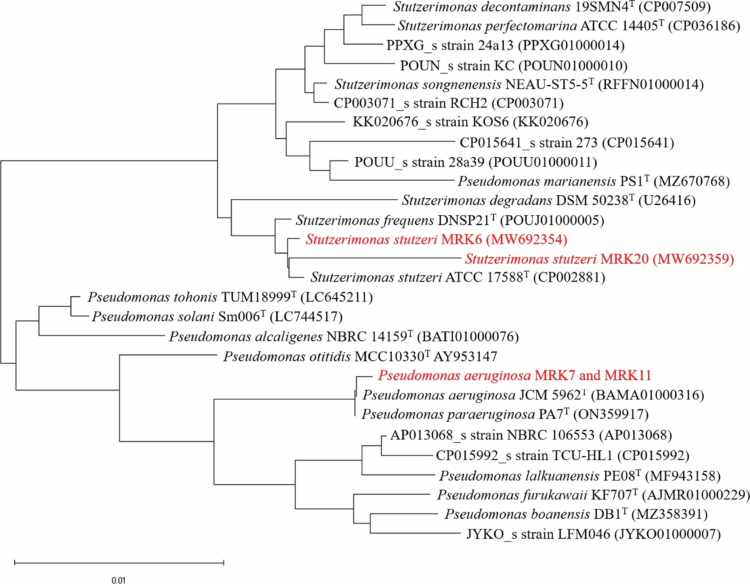
The neighbor-joining phylogenetic tree based on 16S rRNA gene sequences showed phylogenetic relationships of identified saline environmental strains *S. stutzeri* strains MRK6 and MRK20, and *P. aeruginosa* MRK7 and MRK11 (highlighted in red text) with their closely related type strains retrieved from the EzBioCloud database; this tree illustrates the evolutionary relatedness among the strains, with *S. stutzeri* MRK6 and MRK20 showing distinct phylogenetic positions, while *P. aeruginosa* MRK7 and MRK11 showed identical clustering based on 16S rRNA sequence similarity.

The best-performing halophilic bacterial strains, *viz*. MRK6, MRK7, MRK11, and MRK20 were selected based on the highest salt tolerance, plant growth promotion, and having multiple beneficial traits. These strains were identified as *Stutzerimonas stutzeri* MRK6, *Pseudomonas aeruginosa* MRK7, *P. aeruginosa* MRK11, and *S. stutzeri* MRK20 through 16S rRNA sequencing (Figure 4). These halophilic strains had strong power to solubilize insoluble minerals of tri-calcium phosphate, mica, zinc oxide, and manganese oxide under 100 mM NaCl (Figure 3B–D). These halophilic strains demonstrated the highest MSD, followed by PSD. However, these strains had the highest ZSI and ZSE. *S. stutzeri* MRK6 and *P. aeruginosa* MRK7 had the highest PSD and MSD, while *S. stutzeri* MRK20 also showed the highest MSD. *S. stutzeri* MRK6 had the highest PSI, PSE, MSI, and MSE. *P. aeruginosa* MRK7 had the highest ZSI and ZSE, while maximum KSI and KSE were observed from *P. aeruginosa* MRK11.

### Halophilic bacterial strains promoted wheat growth under salt stress

**Figure 5.**
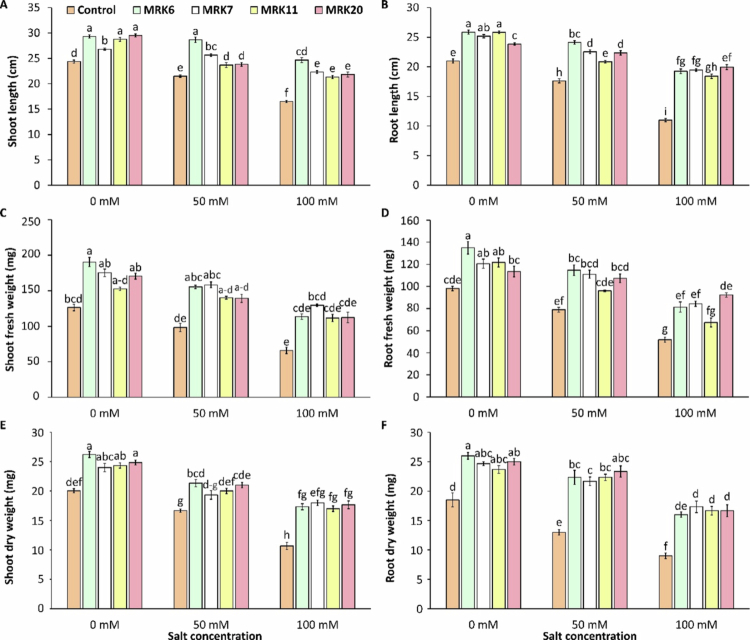
The halophilic bacterial strains promoted wheat seedling growth attributes, including shoot length (a), root length (b), shoot fresh weight (c), root fresh weight (d), shoot dry weight (e), and root dry weight (f) under salt stress; the presented bar graphs of plant growth attributes are mean values of three replications statistically analyzed using factorial design arrangements; the mean values were compared through alphabetical letters and standard error; the bars with same alphabetical letters were considered statistically non-significant at *p* ≤ 0.05.

Salt stress significantly reduced wheat growth, whereas halophilic bacterial strains positively affected it under salt stress (Figure 5). Shoot length and root length decreased by 12% and 16% in 50 mM and 32% and 48% in 100 mM salt stress compared to 0 mM. *S. stutzeri* MRK6 increased shoot length by up to 33% in 50 mM and 49% in 100 mM salt stress compared to the uninoculated control of respective salt stress. It increased root length (37%) in 50 mM salt stress, while *S. stutzeri* MRK20 increased the root length (81%) under 100 mM salt stress over the uninoculated control. The reductions in shoot fresh weight and root fresh weight were up to 48% and 47%, respectively, under 100 mM salt stress compared to the respective uninoculated controls. *P. aeruginosa* MRK7 promoted shoot fresh weight up to 96% in 100 mM salt stress over the respective uninoculated control. The increase in root fresh weight up to 78% at 100 mM was due to *S. stutzeri* MRK20. *S. stutzeri* MRK6 promoted shoot dry weight with an increase of 28% over the uninoculated control. In comparison, the highest root dry weight, with an increase of 72% under 50 mM salt stress, was observed from *S. stutzeri* MRK20 compared to the uninoculated control. Under 100 mM salt stress, the applied halophilic strains had significantly higher shoot and root dry weights than the uninoculated control. The reduction in shoot and root dry weight due to the uninoculated control was up to 17% and 30% in 50 mM and 47% and 51% in 100 mM compared to without salt stress.

### Halophilic bacterial strains promoted wheat physiological attributes under salt stress

**Figure 6.**
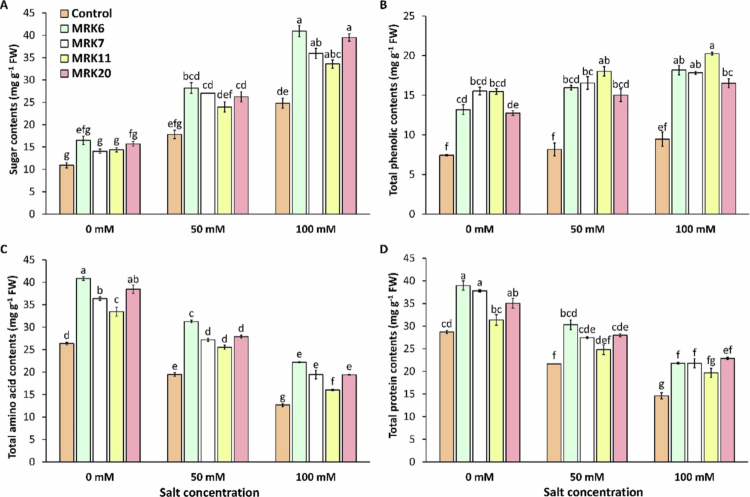
Halophilic bacterial strains promoted sugar content (a), phenolic contents (b), amino acid (c), and protein contents (d) in wheat seedling leaves under salt stress; the presented bar graphs of osmolytes and protein contents are mean values of three replications statistically analyzed using factorial design arrangements; the mean values were compared through alphabetical letters and standard error; the bars with same alphabetical letters were considered statistically non-significant at *p* ≤ 0.05.

**Figure 7.**
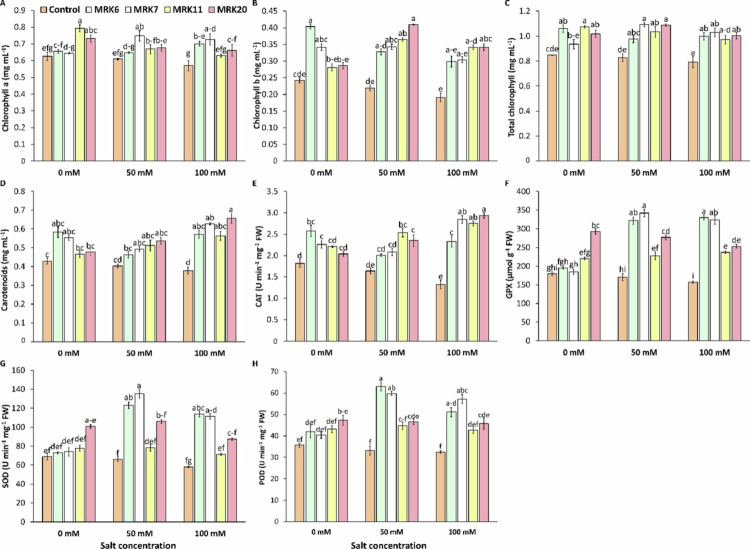
Halophilic bacterial strains promoted chlorophyll, including chlorophyll a (a), chlorophyll b (b), total chlorophyll (c), and carotenoid contents (d) and antioxidant enzymes, including catalase (CAT; e), glutathione peroxidase (GPX; f), superoxide dismutase (SOD; g), and peroxidase (POD; h) in wheat seedling leaves under salt stress; the presented bar graphs of chlorophyll contents and antioxidant enzymes are mean values of three replications statistically analyzed using factorial design arrangements; the mean values were compared through alphabetical letters and standard error; the bars with same alphabetical letters were considered statistically non-significant at *p* ≤ 0.05.

Increasing salt stress raised sugar and phenolic contents in the wheat leaves (Figure 6a and b). The uninoculated control had 63% and 10% higher sugar and phenolic contents in 50 mM and 127% and 22% higher sugar and phenolic contents in 100 mM salt stress compared to 0 mM. *S. stutzeri* MRK6 reported the highest sugar contents with a rise to 58% in 50 mM and 65% in 100 mM salt stress compared to the respective uninoculated control. The highest phenolic contents (114% increase) were observed from *P. aeruginosa* MRK11-treated wheat seedlings in 100 mM stress over the respective uninoculated control. We observed a reduction in amino acid and protein contents of up to 52% and 49% in 100 mM Compared to without salt stress (Figure 6C and D). However, *S. stutzeri* MRK6 promoted amino acid contents of up to 75% in 100 mM over without salt stress. It also showed higher 40% protein contents in 50 mM salt stress compared to the uninoculated control. In 100 mM salt stress, a 57% rise in protein contents was observed due to inoculation with *S. stutzeri* MRK20 over uninoculated control. The salt stress caused a substantial reduction in the chlorophyll and carotenoid contents (Figure 7A–D). The uninoculated control reported a decrease in chlorophyll a by 3% and 9%, chlorophyll b by 10% and 21%, total chlorophyll contents by 3% and 7%, and carotenoids by 5% and 12% in 50 mM and 100 mM, respectively, compared to without salt stress. *P. aeruginosa* MRK7 alleviated salt stress by promoting chlorophyll a, with an increase of 23% in 50 mM and 27% in 100 mM salt stress compared to the respective uninoculated control. The highest increase in chlorophyll b of 67% and 87% in 50 mM and 79% and 80% in 100 mM salt stress was due to *P. aeruginosa* MRK11 and *S. stutzeri* MRK20, over the uninoculated control. *P. aeruginosa* MRK7 and *S. stutzeri* MRK20 had the highest rise in total chlorophyll contents, up to 32% and 31% in 50 mM and 30% and 27% in 100 mM salt stress, compared to the uninoculated control. The increase in carotenoid contents was 33% in 50 mM and 74% in 100 mM salt stress because *S. stutzeri* MRK20 compared to the uninoculated control.

### Halophilic bacterial strains promoted antioxidant enzymes in wheat under salt stress

The antioxidant enzyme activities declined with increased salt stress. The decline in the CAT and GPX of the uninoculated control was 10% and 5% in 50 mM and 27% and 13% in 100 mM Compared to without salt stress (Figure 7E and F). However, plant growth-promoting halophilic bacterial strains boosted these antioxidant enzymes and had higher activity under salt stress. Under 100 mM salt stress, the increase in CAT activity was 123% due to *S. stutzeri* MRK20 compared to the uninoculated control, while in 50 mM salt stress, the increase in CAT activity was 55% due to *P. aeruginosa* MRK11 Compared to its uninoculated control. *S. stutzeri* MRK6 and *P. aeruginosa* MRK7 had the highest GPX activity in wheat leaves, with increases of 88% and 110% in 50 mM salt stress and 100% and 106% in 100 mM salt stress Compared to without salt stress. The increase in salt stress caused a reduction in SOD activity by 4% in 50 mM and 15% in 100 mM, and POD activity by 7% in 50 mM and 9% in 100 mM, as Compared to without salt stress (Figure 7G and H). *S. stutzeri* MRK6 and *P. aeruginosa* MRK7 inoculation promoted SOD activity by 86% and 104% in 50 mM and 96% and 92% in 100 mM, compared to the uninoculated control. These strains also demonstrated the highest increase in POD activity by 90% and 80% in 50 mM and 58% and 76% in 100 mM over their respective uninoculated control.

### Influence of halophilic bacterial strains on nutrient contents under salt stress

**Figure 8.**
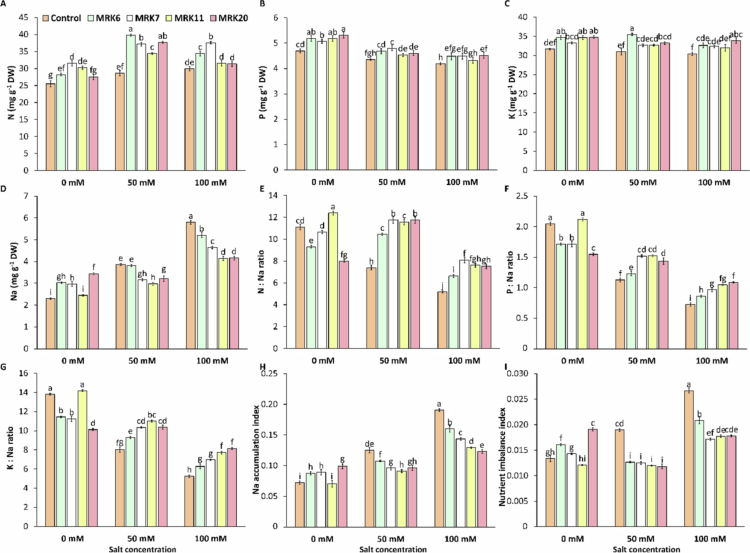
Ion homeostasis in wheat seedlings observed in terms of nitrogen (N; a), phosphorus (P; b), potassium (K; c), sodium (Na; d), N:Na ratio (e), P:Na ratio (f), K:Na ratio (g), Na accumulation index (h), and nutrient imbalance index (i) in wheat seedling influenced by halophilic bacterial strains under salt stress; the presented bar graphs are mean values of three replications statistically analyzed using factorial design arrangements; the mean values were compared through alphabetical letters and standard error; the bars with same alphabetical letters were considered statistically non-significant at *p* ≤ 0.05.

Salt stress disturbed the nutrient concentration in the wheat seedlings, while halophilic bacterial strains was alleviated salt stress by increasing nutrient uptake (Figure 8). These strains upregulated N content by up to 39% with *S. stutzeri* MRK6 under 50 mM salt stress, while *P. aeruginosa* MRK7 had a 26% more N content than the uninoculated control. The halophilic bacterial strains overcome this reduction in P and K contents in wheat seedlings under salt stress. *P. aeruginosa* MRK7 improved the P contents by 10%, while *S. stutzeri* MRK20 had 8% more P contents than the respective uninoculated control under 100 mM salt stress. Strain MRK20 had 11% higher K contents than the uninoculated control in wheat seedlings grown under 100 mM salt stress. *S. stutzeri* MRK6 demonstrated 14% higher K contents over uninoculated control in wheat seedlings grown in 50 mM salt stress. The increase in salt stress demonstrated higher Na contents, Na accumulation index, and nutrient imbalance index, and lower N:Na, P:Na, and K:Na ratios. However, halophilic bacterial strains reduce the Na contents and ion imbalance by increasing the N:Na, P:Na, and K:Na ratios. *P. aeruginosa* MRK11 and *S. stutzeri* MRK20 showed the highest reduction of 29% and 28% in Na contents and 32% and 35% in Na accumulation index over uninoculated control in wheat seedlings grown in 100 mM salt stress. The highest reduction in the nutrient imbalance index, up to 36% compared to uninoculated control, was observed from *P. aeruginosa* MRK7-treated wheat seedlings grown in 100 mM salt stress. The highest N:Na ratio was 57% in 100 mM due to *P. aeruginosa* MRK7 compared to the uninoculated control. *S. stutzeri* MRK20-treated wheat seedlings grown under 100 mM salt stress showed a 50% increase in the P:Na ratio and a 55% increase in the K:Na ratio compared to uninoculated controls.

### Production of organic acids and their role in sodium toxification

**Figure 9.**
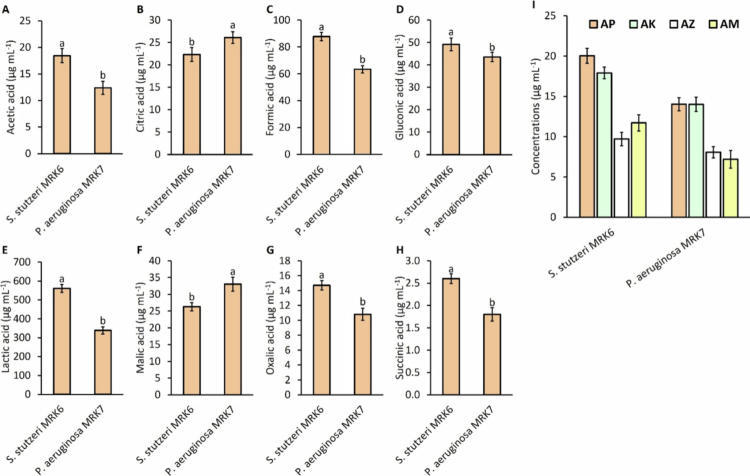
The production of organic acids through halophilic bacterial strains in response to consortium of insoluble minerals under 100 mM NaCl stress; these halophilic bacterial strains produced organic acids, including acetic acid (a), citric acid (b), formic acid (c), gluconic acid (d), lactic acid (e), malic acid (f), oxalic acid (g), and succinic acid (h) and promoted available phosphorus, potassium, zinc, and manganese (i) under salt stress; the organic acids concentrations represent the final net production and these values were quantified after correction against the corresponding sample blanks/negative controls during data processing.

The halophilic bacterial strains increased the availability of nutrients by solubilizing a consortium of insoluble minerals through the production of organic acids (Figure 9). They produced the highest concentration of lactic acid, followed by formic acid and gluconic acid, in response to a consortium of insoluble minerals under salt stress. *S. stutzeri* MRK6 showed stronger organic acid-mediated nutrient mobilization by producing acetic acid (49%), formic acid (38%), gluconic acid (13%), oxalic acid (36%), succinic acid (44%), and lactic acid (66%) compared to *P. aeruginosa* MRK7. These results indicated a dominant acidogenic phenotype linked to mineral dissolution by *S. stutzeri* MRK6. These organic acids produced by halophilic bacterial strains were connected with the increase in nutrient availability, as *S. stutzeri* MRK6 increased available phosphorus by 43%, potassium by 28%, zinc by 20%, and manganese by 61% compared to *P. aeruginosa* MRK7 in response to a consortium of insoluble minerals under salt stress.

### Key physiological traits driving salt tolerance in wheat attributes

**Figure 10.**
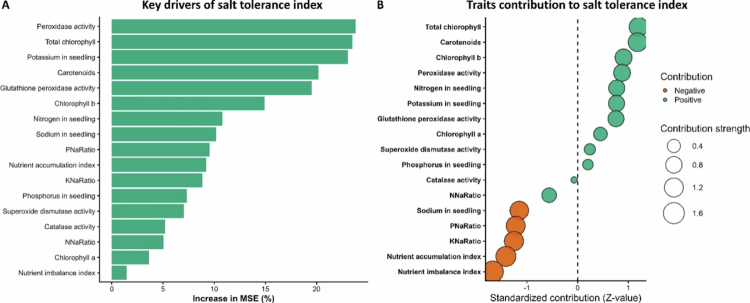
The key physiological drivers associated with salt tolerance in wheat; (a) random forest-based ranking of key physiological drivers of the salt tolerance index; higher values indicate stronger contribution to the salt tolerance index; (b) positive and negative contribution patterns of physiological traits associated with the salt tolerance index; positive values indicate traits were positively associated with increase in salt tolerance, whereas negative values indicate inverse associations; bubble size represents the magnitude of contribution strength, and color indicates the direction of association.

Random forest regression revealed key physiological traits contributing to the salt tolerance index in wheat seedlings (Figure 10A). The peroxidase activity, total chlorophyll content and potassium in seedlings showed the strongest predictor of salt tolerance. The other highly influential traits were carotenoid accumulation, glutathione peroxidase activity, and chlorophyll b contents. The dominance of antioxidant enzymes and photosynthetic traits among the key drivers showed maintenance of the cellular redox balance and photosynthesis represent major components underlying improved salt tolerance. The potassium concentration also showed high predictive importance because of its central role of potassium homeostasis in maintaining physiological stability under sodium-induced stress. Trait contribution analysis revealed clear positive and negative directional associations between physiological traits and the salt tolerance index (Figure 10B). Chlorophyll contents and antioxidant-related traits showed predominantly positive relationships with salt tolerance, indicating their role in wheat seedling performance under saline conditions. In contrast, traits associated with ionic imbalance and stress accumulation showed negative relationships with salt tolerance index. Sodium accumulation showed an opposite contribution pattern, consistent with the detrimental effects of excessive sodium uptake on cellular processes. These results indicate that salt tolerance in the studied bacterial treatments is associated with enhanced antioxidant protection, chlorophyll contents, and improved nutrient‒ion equilibrium.

## Discussion

### Halophilic bacterial strains possess multiple plant growth-promoting traits

**Figure 11.**
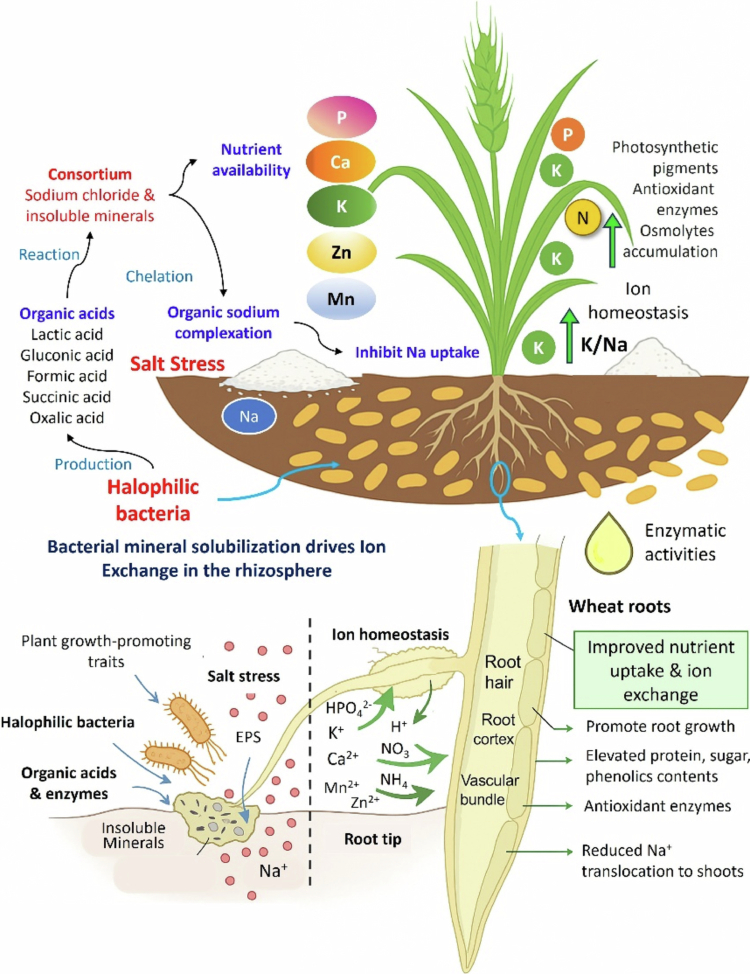
The concept of halophilic bacterial strains in mitigating salt stress in wheat; these halophilic bacterial strains promoted enhanced nutrient availability and plant metabolism through dissolution of insoluble compounds and enzymatic activities; they also produce beneficial metabolites, including exopolysaccharides that improve soil structure, ammonia for nitrogen supply, hydrogen cyanide for pathogen suppression, and siderophores that facilitate iron acquisition; their ability to fix atmospheric nitrogen provides a sustainable source of nitrogen for plant use; mutually, these traits in halophilic bacteria are involved in alleviation of salt stress in wheat by boosting plant growth, chlorophyll contents, osmolytes accumulation, antioxidant enzymatic activities, and ion homeostasis; halophilic bacterial strains maintain osmotic balance and protect cells from dehydration and oxidative damage by stimulate the accumulation of osmolytes such as sugars, phenolics, proteins, amino acids, and carotenoids; they overcome salt stress by neutralizing reactive oxygen species through their antioxidant enzymes; a crucial aspect of halophilic bacterial strain functions is the regulation of ion homeostasis, which reduces Na^+^ toxicity while promoting the uptake of essential nutrients.

In this study, bacterial strains demonstrated their halophilic nature at high salt levels up to 2.0 M NaCl stress. These bacteria may have adaptive mechanisms for enduring high salt stress, including the accumulation of compatible solutes, the regulation of ion balance in their cells, and the overexpression of stress response proteins.^47^ ^,^ ^48^ We observed the highest salt tolerance in *P. aeruginosa* strains MRK7 and MRK11 up to 2.0 M NaCl stress. Previous studies also reported such threshold levels of salt stress for halophilic microorganisms isolated from coastal regions, saline soil, and hypersaline lakes.^49–51^ In the current study, we isolated halophilic bacterial strains from the saline soil and brine pond of the Khewra salt mine, Pakistan, for their potential application in alleviating salt stress in cereal crops. Similarly, Babar et al.^52^ and Haroon et al.^53^ also screened halophilic bacteria from the Khewra salt mine using salt stress levels of 1.0 M and 0.34 M, respectively, to overcome salt stress in wheat, which is lower than the salt stress tested in the current study. The exact mechanisms performed by the halophilic bacterial strains of the current study warrant further investigation to elucidate the genetic and metabolic pathways involved in salt stress adaptation and explore their potential industrial application. The mechanism of action performed by halophilic bacteria under salt stress is proposed and illustrated in Figure 11.

In this study, NaCl stress tolerance was tested by growing bacterial strains in LB medium. Although LB medium is a commonly used medium for culturing diverse bacterial taxa, including *Pseudomonas* and *Stutzerimonas*, it contains complex organic components such as yeast extract and casein peptone. These components are rich in amino acids such as proline and glutamic acid, which are well-known compatible solutes that can be taken up by bacteria to mitigate osmotic stress.^54^ These solutes may partially boost the intrinsic salt tolerance mechanisms of the tested halophilic bacterial strains, which can lead to an overestimation of their saline stress tolerance. Owing to this limitation, we plan to perform future experiments with the use of defined minimal media without organic nutrients to test stress tolerance in beneficial bacterial strains. This approach allows research to precisely evaluate the physiological and metabolic responses of halophilic bacteria to abiotic stress and can validate bacterial effectiveness as bioinoculants under extreme conditions.

Halophilic bacteria have a potential biotechnological application in hypersaline environments and may contribute to nutrient cycling and ecosystem functioning. In this study, the tested halophilic bacterial strains exhibited multiple plant growth-promoting traits, including indole compound production under 100 mM NaCl, N_2_-fixation, production of siderophore, ammonia, and HCN. These traits are beneficial for plant growth promotion under normal conditions and even under salt stress. Like our bacterial potential, Babar et al.^52^ and Haroon et al.^53^ also reported halophilic bacterial strain's ability to produce phytohormones, siderophores, HCN, ammonia, and N_2_-fixation, which contributed to plant growth promotion. Additionally, hydrolysis of starch and the utilization of citrate by our halophilic bacteria align with Reang et al. ^55^, who also reported the ability of their halophilic bacterial strains to utilize various other substrates, including glucose, adonitol, lactose, arabinose, sorbitol, lysine, and ornithine. Utilizing such diverse substrates may also be helpful for our halophilic bacterial strains to adjust and survive in hypersaline conditions. The EPS production by our halophilic bacterial strains may have a crucial role in attenuating salt stress through binding soil particles and forming biofilms around bacterial cells and plant roots. Shahid et al.^56^ reported that halotolerant EPS-producing bacteria promoted salt tolerance by acting as a protective barrier around plant roots. The EPS reduces the toxic effect of salt ions and keeps the plant root cells hydrated even under salt-stress conditions.^57^ The enzymatic activities of our tested halophilic bacteria, including amylase, catalase, cellulase, lipase, protease, oxidase, and urease activities, demonstrated their metabolic versatility and contribution toward nutrient cycling by degrading organic matter.

In the present study, halophilic *S. stutzeri* (strains MRK6 and MRK20) and *P. aeruginosa* (strains MRK7 and MRK11) demonstrated dissolution of phosphate, mica, zinc oxide, and manganese oxide under 100 mM NaCl. These strains exhibited the highest MSD under 100 mM NaCl. In contrast, the halophilic bacterial strains showed maximum ZSI, KSI, ZSE, and KSE under 100 mM NaCl. The mineral dissolution by our halophilic bacterial strains might be due to organic acid production, chelating agents, and enzymatic activities. These mechanisms by halophilic bacterial strains mediate a reduction in soil pH, facilitating nutrient bioavailability from insoluble mineral complexes.^58^ Reang et al.^55^ reported dissolution of phosphate and potassium by halophilic *Halomonas pacifica*. Mukhtar et al.^28^, Babar et al.^52^, and Haroon et al.^53^ also reported that phosphate dissolution by halophilic bacterial strains, which produce organic acids that react with insoluble phosphate compounds and convert them into orthophosphate readily available for plant uptake. In the current study, mica dissolution by halophilic bacterial strains may result from acidolysis, through which bacterial acids degrade the mica structure and promote potassium availability.^59^ The dissolution of zinc oxide and manganese oxide by our halophilic bacterial strains highlights their versatility in reducing or chelating metal ions and has a potential role in nutrient availability. Our halophilic bacterial ability to solubilize such nutrients aligns with previous studies.^60–62^

The four best-performing *S. stutzeri* (strains MRK6 and MRK20) and *P. aeruginosa* (MRK7 and MRK11), belong to only two genera. Although they are classified as the same species, differences in their genetic background, metabolic capacity, and environmental adaptation may lead to variability in their plant growth-promoting potential. This is consistent with previous findings, such as those reported by Zeng et al.^63^ and Dadaşoğlu et al.^64^ which demonstrated that intra-species diversity can result in significant functional differences, particularly in traits such as IAA production, phosphate dissolution, or stress tolerance. We acknowledge previous work documenting the plant growth-promoting traits of *S. stutzeri*, including siderophore production, phosphate dissolution, indole compound synthesis, HCN and ammonia production, as well as enzymatic activity.^65^ ^,^ ^66^ Our findings are consistent with these reports and further highlight the salt tolerance and multifaceted PGP potential of the strains evaluated in this study.

### Halophilic bacteria promoted plant growth and osmolyte accumulation under salt stress

In the present study, salt stress caused a reduction in wheat growth, which might be due to osmotic and ionic stresses. Salt stress promotes the accumulation of Na and Cl ions in plant cells, disrupting ion homeostasis, enzyme activities, and nutrient uptake.^4^ It induces osmotic stress by limiting water uptake and cell expansion, leading to dehydration.^67^ In this study, inoculation with halotolerant *P. aeruginosa* strains MRK7 and MRK11 and *S. stutzeri* strains MRK6 and MRK20 significantly counteracts the adverse effects of salt stress by promoting root and shoot growth attributes. These halophilic bacterial strains could produce indole compounds, a key phytohormone that encourages plant growth under salt stress. IAA overcomes osmotic stress in plants by improving root architecture, which promotes water and nutrient uptake under salt stress.^68^ The increase in wheat root growth and root biomass in the present study is correlated with the production of indole compounds with and without L-tryptophan. Gull et al.^69^ reported alleviating salt stress through the exogenous application of IAA and L-tryptophan, indicating IAA's role in promoting root growth under salt stress. Grossi et al. ^70^ reported improved root architecture through auxin-producing *Methylobacterium* sp. 2A. Lu et al.^71^ also reported that salt stress was alleviated through indole-3-carboxaldehyde-producing *Streptomyces lasalocidi* JCM 3373^T^ by shaping root architecture in soybean. Our halophilic bacterial strains possess multiple growth-promoting traits that might have a direct or indirect roles in alleviating salt stress. These traits directly promote nutrient uptake through biological N_2_ fixation, ammonia production, mineral dissolution, and the production of siderophores under salt stress. The production of HCN, EPS, and enzymatic activities might be critical in mitigating oxidative damage due to salt stress. The plant growth promotion due to multiple traits of bacterial strains under salt stress aligns with the results of previous studies.^72–75^

This study revealed that salt stress significantly affects the accumulation of osmolytes, including sugars, total phenolics, total amino acids, and protein contents, which might be adaptive responses by plants to survive in hostile conditions. We observed the increase in the accumulation of total soluble sugar and total phenolic contents, which might help plants sustain water balance in plant cells under salt stress. Similarly, Zarbakhsh and Shahsavar^76^ also reported an increase in sugar and phenolic contents under salt stress, which has a potential role in coping with salt stress. In this study, we observed reduced total amino acid and protein contents under salt stress. Previous studies reported contrasting results regarding total amino acid and protein contents under salt stress. Gilbert et al.^77^ reported increased amino acids for the first 10 d of exposure and then a decline due to salt stress. Hasanuzzaman and Fujita^78^ supported our findings of a reduction in protein content under salt stress. This study demonstrated the mitigation of salt stress by promoting soluble sugar, total phenolic contents, amino acids, and protein contents in wheat seedlings through inoculation with halophilic bacterial strains. Promoting these biochemical attributes could be part of adaptive mechanisms that allow halophilic bacterial strains to encourage plant survival under salt stress. These bacterial strains might produce soluble sugar, phenolic compounds, amino acids, and proteins, which help plants maintain osmotic stability and protect their cellular components. These osmolytes are water-soluble and prevent plasmolysis during salt stress. Our findings align with the results of previous studies.^79–81^

### Halophilic bacteria promoted chlorophyll and antioxidant defense under salt stress

Salt stress negatively affects the photosynthetic process by reducing the total chlorophyll and carotenoid contents in wheat seedlings of uninoculated control. During the photosynthetic process, chlorophyll and carotenoid pigments are crucial for the absorption of sunlight and the transfer of energy, but their reduction under salt stress reduces plant growth. Salt stress inhibits enzymes, e.g., chlorophyll synthase and protochlorophyllide reductase,^82^ and induces ROS production, which degrades chlorophyll and carotenoid molecules.^82^ ^,^ ^83^ In the current study, halophilic bacterial strains mitigated the effects of salt stress by improving chlorophyll in wheat seedlings. These bacteria promote the production of enzymatic and non-enzymatic antioxidants in plants that scavenge ROS, protect chlorophyll, and reduce oxidative damage under salt stress.^84^ ^,^ ^85^ Mohamed and Gomaa^86^ also reported increased chlorophyll a, b, and carotenoid contents by inoculating radish plants with *Bacillus subtilis* and *Pseudomonas fluorescens* under salt stress. The increased photosynthetic process under salt stress was also achieved through inoculation with *Bacillus pumilus* JPVS11, *Jeongeuplla avenae* gen., *Gracilibacillus dipsosauri* GDHT17, and *Pseudomonas atacamensis* KSS‐6.^87–89^ In the present study, the increase in chlorophyll was more pronounced in 50 mM NaCl due to effective bacterial mechanisms that counteracted salt stress. Under 100 mM salt stress, the bacterial strains still maintained chlorophyll at significantly higher levels than the uninoculated control. Our findings also align with the previous findings of Galicia-Campos et al.^85^ and Arora et al.^87^

Salt stress disrupts cellular homeostasis, which generates excessive ROS in chloroplasts, mitochondria, and peroxisomes, leading to lipid, protein, and DNA damage.^90^ ^,^ ^91^ Plants survive salt stress based on antioxidant enzymes, including CAT, SOD, POD, and various non-enzymatic antioxidants, which scavenge ROS and promote the plant defense system.^92^ This study demonstrated the higher activities of antioxidant enzymes, including CAT, APX, SOD, and POD, in wheat seedlings treated with halophilic bacterial strains, which enable plants to cope with the oxidative stress induced by salt stress. Sagar et al.^93^ reported the antioxidant enzyme-producing ability of halophilic *Enterobacter* sp. PR14, which might be true in the case of our halophilic bacterial strains. The antioxidant enzymes produced by the halophilic *P. aeruginosa* strains MRK7 and MRK11 and *S. stutzeri* strains MRK6 and MRK20 might directly scavenge ROS in the rhizosphere and reduce oxidative stress in plant roots. The plant can absorb these bacterial antioxidants and could be involved in the stimulation of endogenous antioxidant enzyme production. Wheat seedling inoculation with halophilic bacterial strains could upregulate the expression of the *cat* gene for catalase, the *apx* gene for glutathione peroxidase, the *sod* gene for superoxide dismutase, and *pod* gene for peroxidase enzyme production in plants under salt stress, as previously reported by Hu et al.^94^ and Wang et al.^95^ The elevated SOD activity in plants catalyzes the dismutation of superoxide anion into oxygen and H_2_O_2._ ^96^ Plant microbial inoculation promotes CAT, GPX, and POD activity, which overcomes oxidative damage due to salt stress by decomposing H_2_O_2_ into H_2_O and O_2._ ^97^ These antioxidant enzymes protect cellular integrity and maintain redox homeostasis.^98^

### Halophilic bacteria promoted ion homeostasis and salt stress tolerance

Salt stress in uninoculated control revealed significant disruption in ion homeostasis. We observed the accumulation of Na^+^ in wheat seedlings, which interfered with the uptake of essential nutrients such as K^+^, N, and P under salt stress. This imbalance in ion homeostasis may lead to ionic toxicity, impair wheat growth, and reduce enzyme activity and various metabolic processes. Plant cells counteract salt stress by actively pumping Na^+^ out of the cells through Na^+^/H^+^ antiporter transport systems to reduce intracellular toxicity.^99^ Further, high-affinity K^+^ transporters facilitate K^+^ accumulation to balance osmotic potential and sustain enzymatic activities.^100^ ^,^ ^101^ In this study, halophilic *P. aeruginosa* and *S. stutzeri* promoted wheat seedlings under salt stress by employing ion homeostasis. Wheat seedlings treated with these halophilic bacterial strains reduced Na^+^ uptake, Na-accumulation index, and the nutrient imbalance index under salt stress. This reduction in Na^+^ uptake might be due to the biosynthesis of EPS by our tested halophilic bacterial strains. The microbial EPS and proteins might sequester Na^+^ ions in the rhizosphere and prevent them from plant uptake. This binding is not strong due to the high solubility and diffusibility of Na^+^. However, bacterial and plant proteins also indirectly manage Na^+^ by controlling its transport and sequestration. Liu et al.^102^ reported the binding of EPS from *Pseudomonas simiae* MHR6 with Na^+^ and improved ion homeostasis in maize under salt stress, which might be true in this study for ion homeostasis in wheat.

In this study, halophilic bacterial strains promoted the uptake of N, P, and K^+^, the N:Na ratio, the P:Na ratio, and the K^+^:Na^+^ ratio under salt stress. The increase in these attributes indicates the halophilic bacterial strain's ability to promote ion homeostasis under salt stress. Our tested halophilic bacterial strains may have adopted various approaches, including their ability for biological N_2_-fixation and mineral dissolution, to encourage ion homeostasis under salt stress. Feng et al.^103^ reported enhanced salt stress tolerance in maize through K^+^-solubilizing bacterial strains by improving K+ uptake. Enhanced K^+^ uptake is vital for plants because of its critical role in plant physiology in mitigating salt stress by maintaining ionic balance.^104^ It maintains ionic balance by competing with excessive Na^+^ uptake under salt stress. Bacterial cells sequester Na^+^ and may release various organic acids and H^+^ ions into the rhizosphere, promoting K^+^ availability.^59^ Bacterial inoculation in plants regulates the expression of high-affinity K^+^ transporters to encourage K^+^ uptake under salt stress.^105^ It also mitigates salt stress by suppressing Na^+^ influx through non-selective cation channels and promotes cation channel specificity for K^+^ by protecting signaling molecule production^106^. Nawaz et al.^107^ reported the contribution of K-solubilizing bacteria in K^+^:Na^+^ homeostasis under salt stress, similar to our current findings, as our tested halophilic bacterial strains were well capable of solubilizing K^+^ and also reported the increase in K^+^:Na^+^ ratio under salt stress. Similarly, Tang et al.^108^ reported increased K^+^:Na^+^ homeostasis in maize under salt stress through inoculation with salt-tolerant bacteria. The increased K^+^:Na^+^ homeostasis under salt stress by our halophilic bacteria aligns with the findings of Ning et al.^109^ This study highlights that halophilic *P. aeruginosa* (strains MRK7 and MRK11) and *S. stutzeri* (strains MRK6 and MRK20) promoted K^+^ uptake and ensured K^+^:Na^+^ homeostasis by limiting Na^+^ uptake and accumulation in plants under salt stress. These bacterial strains upregulated the scavenging of ROS by producing antioxidant enzymes and boosted plant growth through elevated osmolyte accumulation and photosynthetic processes under salt stress.

Although the present study demonstrated the functional potential of halotolerant bacterial strains in improving plant salt stress tolerance, future investigations integrating molecular-based approaches and advanced metabolite profiling will be required to elucidate the underlying molecular mechanisms of these interactions. Although the *P. aeruginosa* strain used in this study showed strong plant growth-promoting traits and increased salinity tolerance in wheat seedlings, we acknowledge that its potential biosafety concerns require careful consideration. The current work did not include molecular analyses for the detection of virulence-associated genes, such as the phenazine biosynthesis (*phz*) gene and other pathogenicity-related factors. A careful biosafety assessment and comprehensive molecular screening are necessary in future studies to evaluate such a potential strain for agricultural applications to eliminate potential risk.

## Conclusions

The halophilic bacteria possess multifunctional broad-spectrum plant growth-promoting traits, having the ability to solubilize minerals, nitrogen fixation, production of indole compounds, siderophores, HCN, and enzymatic activities. They increase wheat plant growth, chlorophyll and carotenoid contents, accumulation of sugars, proteins, phenolics, and amino acids, antioxidant defense mechanisms, and ionic balance by increasing N, P, and K uptake and reducing Na accumulation. Halophilic *S. stutzeri* MRK6 was a potential bioinoculant and offers a sustainable solution for improving crop resilience under salt stress. Although the identified *P. aeruginosa* strains demonstrated beneficial effects under controlled conditions, their potential biosafety concerns require further genomic and pathogenicity assessments before considering any agricultural application. This study was conducted under controlled conditions, but field trials under natural saline conditions must be undertaken to validate the findings practically. The underlying genomic, transcriptomic, and regulatory pathways of these halophilic bioinoculants under salt stress must also be investigated.
